# Anti-NMDAR encephalitis following malaria: expanding the spectrum of post−malarial neurological syndromes

**DOI:** 10.3389/fimmu.2026.1762504

**Published:** 2026-03-12

**Authors:** Shuwen Deng, Wenlong Wang, Wei Lu, Shige Wang

**Affiliations:** 1 Department of Health Management, The Third Xiangya Hospital, Central South University, Changsha, China; 2 Postdoctoral Station of Clinical Medicine, the Third Xiangya Hospital, Central South University, Changsha, China; 3 Department of Infectious Diseases, The Second Xiangya Hospital, Central South University, Changsha, China; 4 Department of Neurology, The Second Xiangya Hospital, Central South University, Changsha, China; 5 The Second Xiangya Hospital, Central South University, Changsha, China; 6 School of Biological Science, Faculty of Biology, Medicine and Health, University of Manchester, Manchester, United Kingdom

**Keywords:** anti-NMDAR encephalitis, autoimmune encephalitis (AIE), neuroimmunology, *Plasmodium falciparum* malaria, post-malarial neurological syndrome

## Abstract

**Background:**

Anti-N-methyl-D-aspartate receptor (anti-NMDAR) encephalitis is the most common form of autoimmune encephalitis (AIE), often triggered by viral infections or tumors. This report describes a rare case following a parasitic infection.

**Case findings:**

We report a rare case of anti-NMDAR encephalitis developing in a 40-year-old man following recovery from Plasmodium falciparum malaria. Despite confirmed parasite clearance, he presented eleven days later with seizures, altered consciousness, and acute psychosis. Anti-NMDAR antibodies were detected in the cerebrospinal fluid (CSF) via both immunofluorescence and cell-based assay, with no evidence of concurrent infection or malignancy. The patient showed rapid clinical improvement after first-line immunotherapy.

**Conclusion:**

This report represents the first antibody-confirmed case of anti-NMDAR encephalitis following Plasmodium falciparum infection, highlighting malaria as a novel post-infectious trigger and expanding the recognized spectrum of post-malarial neurological syndromes (PMNS). It underscores that clinicians in endemic regions should maintain a high index of suspicion for AIE in patients with new neuropsychiatric symptoms after malaria and pursue early antibody testing and immunotherapy.

## 1 Introduction

Malaria remains one of the most important parasitic diseases worldwide, contributing substantially to morbidity and mortality, particularly in tropical and subtropical regions. Among its neurological manifestations, cerebral malaria is the most severe, frequently leading to seizures, coma, and long-term cognitive sequelae (1). Beyond the acute phase, accumulating evidence has identified post-malarial neurological syndrome (PMNS), a delayed immune-mediated disorder that typically develops within weeks after parasite clearance (2). PMNS is defined as a discrete, self-limited constellation of neurological symptoms emerging after recovery from acute malaria infection and comprises four principal phenotypes: the “classic” PMNS form (seizures, confusion, or psychosis), delayed cerebellar ataxia (DCA), acute inflammatory demyelinating polyneuropathy (AIDP), and acute disseminated encephalomyelitis (ADEM) (3–8). In addition, malaria has been linked to other immune-mediated central nervous system demyelinating disorders, including multiple sclerosis (MS) (9, 10) and neuromyelitis optica spectrum disorder (NMOSD) (11), further supporting the concept of post-infectious autoimmunity in the central nervous system (CNS).

Recent literature has broadened the spectrum of malaria-associated neurological disease beyond PMNS, indicating that specific, antibody-mediated autoimmune encephalitides can occur as post-infectious complications. Encephalitis associated with voltage-gated potassium channel (VGKC)-complex antibodies (12) and anti-septin complex antibodies has been reported after *Plasmodium falciparum* infection, suggesting that malaria may trigger adaptive immune responses against neuronal antigens (13). Collectively, these observations imply that malaria can precipitate VGKC-complex and septin-complex encephalitides and, as we demonstrate in this report, anti-N-methyl-D-aspartate receptor (NMDAR) encephalitis.

Anti-NMDAR encephalitis is a well-characterized and common autoimmune encephalitis (AIE), typically presenting with psychiatric symptoms, seizures, dyskinesias, and autonomic dysfunction. It is classically linked to tumors (e.g., ovarian teratomas) and may also follow viral infections, particularly herpes simplex virus (HSV) encephalitis (14). Pathogen-associated cases are thought to arise through molecular mimicry, bystander activation, and blood-brain barrier (BBB) disruption, which subsequently lead to the exposure of neuronal epitopes and promote antibody production.

To date, no antibody-confirmed case of anti-NMDAR encephalitis triggered by malaria has been reported. To our knowledge, we present the first case of anti-NMDAR encephalitis following P. falciparum malaria with antibody confirmation via both immunofluorescence assay (IFA) and cell-based assay (CBA) and tissue-based assay (TBA). The clear temporal association, absence of other triggers, and favorable response to immunotherapy strongly suggest a post-malarial autoimmune mechanism. This case provides valuable insight into the expanding landscape of post-infectious neuroimmunological syndromes and highlights the need for increased diagnostic vigilance in malaria-endemic areas.

## 2 Case presentation

### 2.1 Initial presentation and neurological onset

The patient was a 40-year-old male who had recently traveled to Africa. He initially presented with symptoms consistent with *Plasmodium falciparum* malaria. Following standard anti-malarial therapy of artesunate and amodiaquine, parasite clearance was confirmed. However, approximately eleven days after initial malarial diagnosis. the patient developed new-onset neuropsychiatric symptoms. These rapidly progressed to include seizures, acute psychosis, and altered consciousness. He was admitted to our hospital later with altered consciousness. Shortly after admission, he experienced another generalized seizure, requiring emergency intervention with diazepam and airway management.

### 2.2 Investigations & laboratory findings: confirmation and exclusion

**Figure 1:**
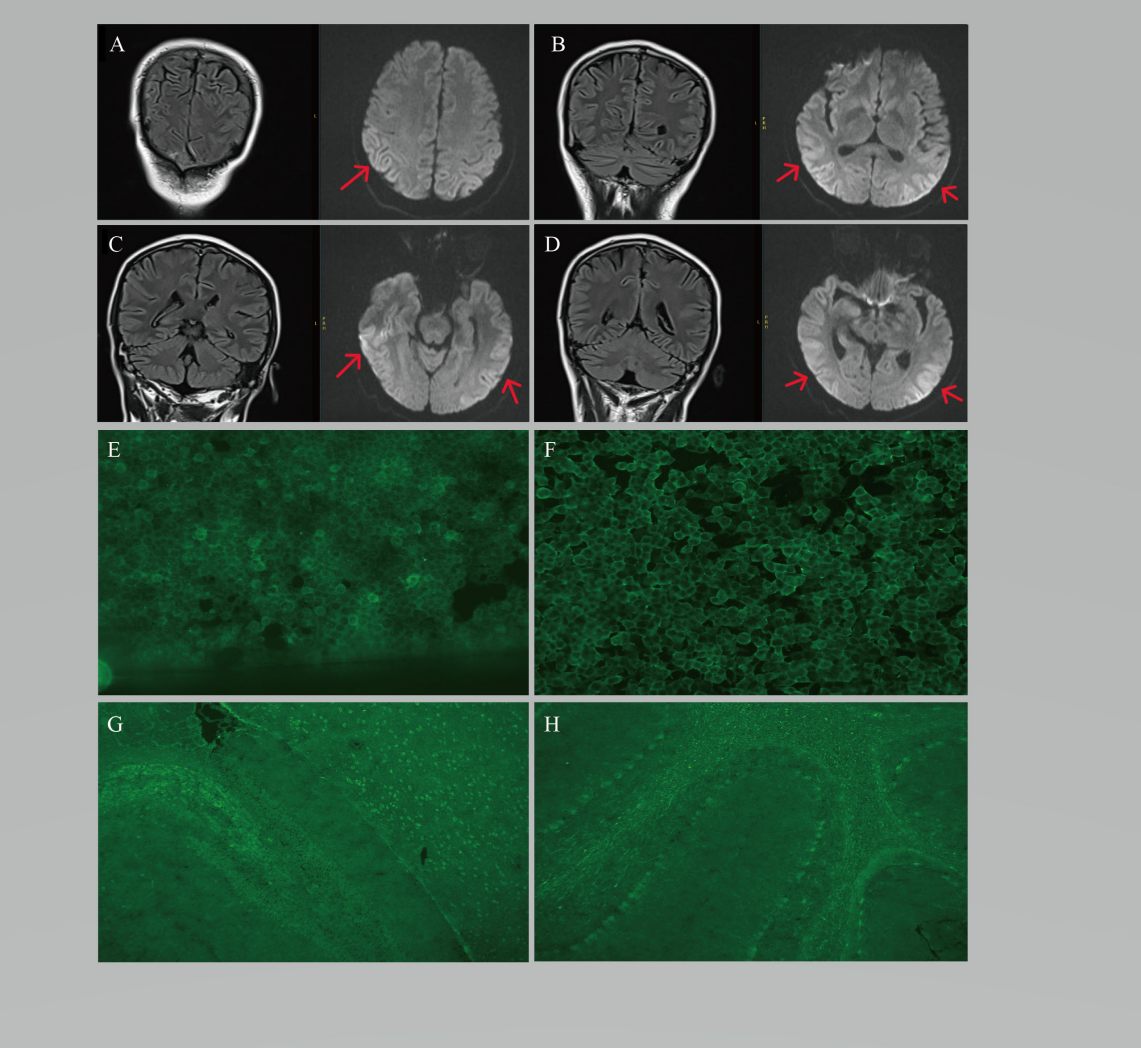
Neuroimaging and laboratory findings of the reported patient with post-malarial anti-NMDAR encephalitis. **(A–D)** Brain MRI sequences showing bilateral temporo-occipital cortical swelling and leptomeningeal enhancement (arrows). **(E)** Cell-based assay (CBA) using the patient’s CSF showing anti-NMDAR IgG binding to transfected cells. **(F)** CBA using paired serum sample showing no specific immunoreactivity. **(G)** Tissue-based assay (TBA) on rodent brain incubated with CSF, demonstrating granular fluorescence in neurons of the corpus callosum, cortex, hippocampus and thalamus. **(H)** TBA on cerebellum showing positive signals in glial cells and neurons within the Purkinje cell layer. The same staining pattern was reproduced in a second CSF sample, consistent with NMDAR autoantibody-associated encephalitis. The corresponding serum sample tested negative (data not shown).

Initial suspicion focused on malaria recrudescence or cerebral malaria, leading to the empirical restart of artesunate. Neurological examination revealed nuchal rigidity, a positive Kernig’s sign and a positive Babinski’s sign. Brain MRI performed on June 5th showed mild bilateral temporo-occipital cortical swelling with leptomeningeal enhancement (**Figure 1**). A lumbar puncture the same day confirmed significant cerebrospinal fluid (CSF) inflammation, with an opening pressure of 220 mmH_2_O, a white blood cell count of 16 × 10^6^/L (lymphocyte-predominant), and an elevated protein level of 743 mg/L. Extensive infectious studies returned negative results. A commercially available cell-based assay for anti-NMDAR antibodies was performed on June 5th. Serum anti-NMDAR IgG was negative, whereas cerebrospinal fluid (CSF) anti-NMDAR IgG was positive with a titer of 1:1 (undiluted). In the same sample, a tissue-based assay (TBA) on rat brain revealed a characteristic neuropil staining pattern: in the cerebrum, granular immunofluorescence involved neurons in the corpus callosum, cortex, hippocampus and thalamus; in the cerebellum, positive signals were observed in glial cells and neurons within the Purkinje cell layer. Concurrently tested antibodies against AMPAR1, AMPAR2, LGI1, CASPR2 and GABAR were all negative. Because the initial CSF NMDAR signal was of low titer, repeat testing was undertaken after 5 days later. CSF anti-NMDAR IgG remained positive with a titer of 1:1 (neat) on CBA, while serum again tested negative. The TBA demonstrated the same distribution of immunoreactivity in cerebral and cerebellar regions as in the first sample, further supporting the presence of NMDAR-targeted autoantibodies. Other neuronal surface antibodies AMPAR1, AMPAR2, LGI1, CASPR2 and GABAR were all remained negative on repeat testing (**Figure 1**).

### 2.3 Treatment and outcome

Following the diagnosis, empirical immunotherapy with intravenous dexamethasone (10 mg daily) was initiated due to the highly suggestive post-infectious encephalitic presentation. The patient exhibited significant and rapid clinical improvement after treatment. A repeat lumbar puncture performed a week after the treatment demonstrated clear therapeutic efficacy, with reduced opening pressure (150 mmH_2_O), WBC count (6 × 10^6^/L), and protein level (554 mg/L). The neuropsychiatric symptoms and seizures subsided, resulting in the complete resolution of symptoms and stabilization of his neurological status. The patient was then discharged with a tapering dose of oral methylprednisolone. At a one-month follow-up, he was asymptomatic with a normal neurological examination. At the most recent follow-up (approximately 8 months after diagnosis), the patient remained asymptomatic with a normal neurological examination and no clinical evidence of relapse. Repeat serum testing for anti-NMDAR IgG at follow-up was negative. The patient provided his written informed consent for the publication of this report.

## 3 Discussion

### 3.1 Potential mechanisms involved in malaria-triggered anti-NMDAR encephalitis

Anti-NMDAR encephalitis is increasingly recognized as an infection-triggered autoimmune disorder, most classically described after herpes simplex virus (HSV) encephalitis. Proposed pathogenic mechanisms include molecular mimicry, bystander immune activation, disruption of immune tolerance, and blood–brain barrier (BBB) dysfunction, which together facilitate exposure of neuronal antigens and intrathecal autoantibody production. While malaria has long been associated with post-infectious neurological syndromes such as post-malarial neurological syndrome (PMNS), antibody-confirmed autoimmune encephalitis following malaria has rarely been documented. To date, no definitive antibody-verified case of post-malarial anti-NMDAR encephalitis has been reported.

**Figure 2:**
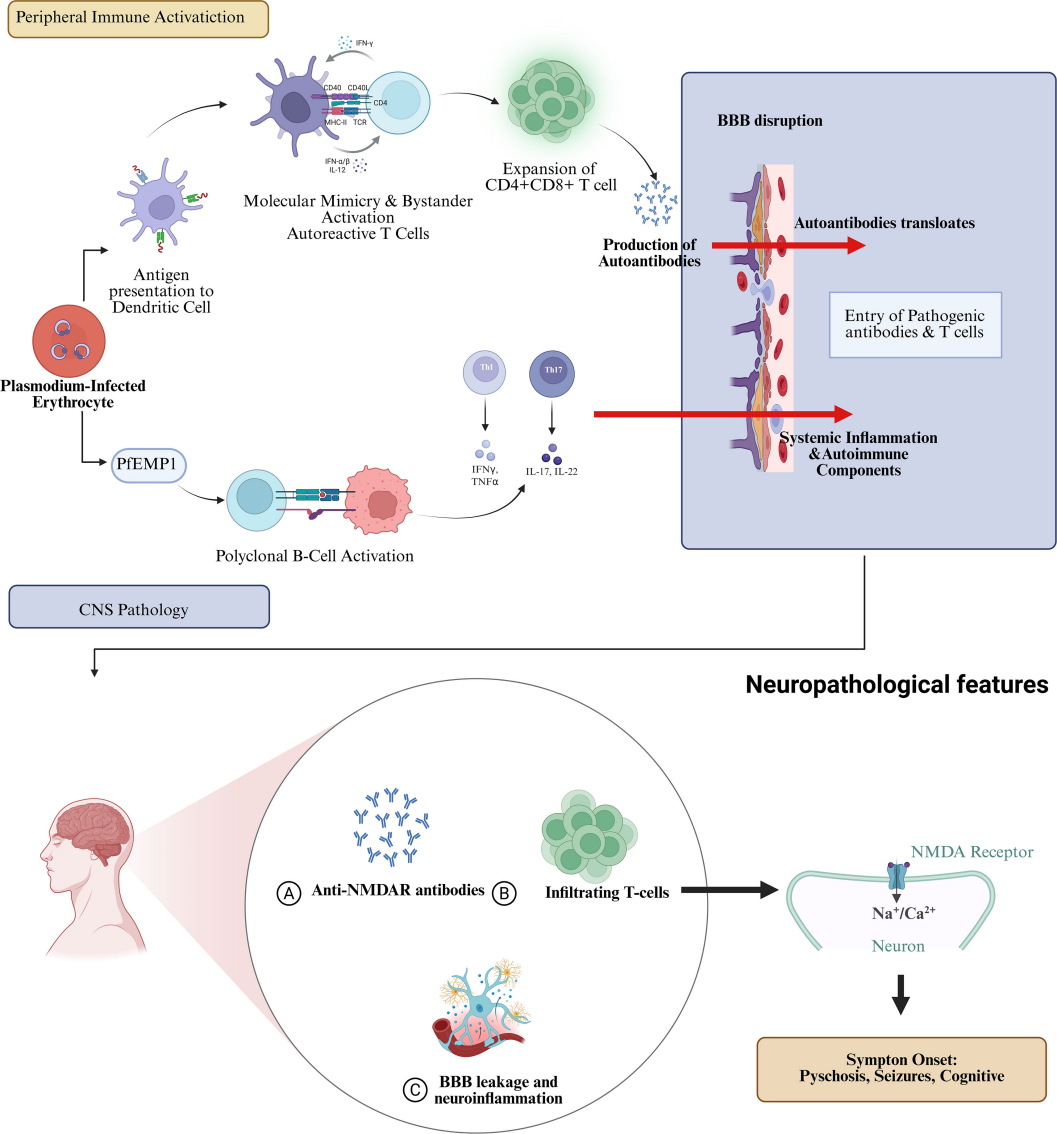
Proposed mechanistic pathways linking malaria infection to anti-NMDAR encephalitis. The process begins with peripheral immune activation during acute malaria (upper), characterized by polyclonal B-cell activation and aberrant T-cell responses. Following parasite clearance, a pro-inflammatory state and loss of immune regulation facilitate the translocation of autoreactive components across the compromised BBB. Within the central nervous system (lower), autoantibodies specifically target NMDARs, leading to neuronal dysfunction and the clinical manifestation of encephalitis.

Based on available experimental and clinical evidence, we propose that malaria-associated anti-NMDAR encephalitis may arise through several non-mutually exclusive immune mechanisms (**Figure 2**).

First, infection-triggered adaptive autoimmunity mediated by molecular mimicry and bystander activation may play a central role. *Plasmodium* infection induces intense systemic inflammation and broad T-cell activation, which may lower the threshold for the expansion of autoreactive lymphocyte. Clinically, several post-malarial neurological disorders mediated by antibodies targeting neuronal surface or synaptic proteins—such as VGKC-complex and septin-complex antibodies—share phenotypic similarities with autoimmune encephalitis, including responsiveness to corticosteroid therapy and reversibility of MRI abnormalities. These observations support an immune-mediated mechanism rather than persistent infection. Although direct identification of malaria-derived mimotopes cross-reactive with NMDAR was not performed in the present case, molecular mimicry remains a biologically plausible hypothesis consistent with post-infectious anti-NMDAR encephalitis observed after other pathogens (15).

Second, polyclonal B-cell overactivation and immunoglobulin skewing may contribute to autoantibody generation. *Plasmodium*-infected erythrocytes express immunoglobulin-binding proteins (IBPs), including PfEMP1, which can persist after parasite clearance and broadly bind circulating immunoglobulins. This interaction may directly stimulate B cells and promote nonspecific antibody production (16). Such immune dysregulation may explain the frequent observation of elevated multivirus IgG/IgM titers, low-titer autoantibodies, cerebrospinal fluid lymphocytic pleocytosis, and intrathecal immunoglobulin synthesis in post-malarial states, even in the absence of concurrent infection. Within this permissive immune environment, pathogenic anti-NMDAR autoantibodies may emerge.

Third, T-cell dysregulation characterized by expansion of CD4^+^CD8^+^ double-positive T cells (DP-T) and a Th1/Th17-skewed inflammatory axis has been demonstrated in experimental *Plasmodium* models. Infection-induced DP-T cells can exacerbate autoimmune neuroinflammation by mounting autoreactive responses to neural antigens and promoting proliferation of encephalitogenic T cells, accompanied by robust production of IFN-γ and IL-17. Although antimalarial agents such as chloroquine can enhance regulatory T-cell responses and dampen experimental autoimmune encephalomyelitis, *Plasmodium* infection may partially override this immunoregulatory effect, potentially through Toll-like receptor-mediated innate immune activation (17). When coupled with transient BBB disruption, these processes may facilitate epitope spreading and sustained intrathecal antibody production.

Finally, dynamic and biphasic immune regulation may influence the timing of autoimmune neurological manifestations. Co-infection models combining malaria and experimental autoimmune encephalomyelitis have demonstrated compartment- and phase-dependent immune modulation, including reduced IL-17A in draining lymph nodes alongside increased IL-10 (18), TGF-β1, and IL-27 and modest expansion of peripheral regulatory T cells (19). Clinically, the weeks following parasite clearance may represent a vulnerable window during which immune rebound and persistent antigen presentation permit the emergence of post-infectious autoimmune encephalitis phenotypes, as observed in the present case.

### 3.2 Expanded public health implications

Although the CSF anti-NMDAR IgG titer was relatively low (1:1, undiluted), the antibody was strictly confined to the CSF on two separate occasions and showed a highly specific neuropil staining in cortex, hippocampus, thalamus and cerebellar Purkinje cells. Together with the characteristic clinical phenotype and robust response to immunotherapy, these findings strongly support a diagnosis of anti-NMDAR encephalitis, rather than nonspecific or incidental antibody positivity.

This case underscores a critical public health message for malaria-endemic regions: malaria can trigger AIE (e.g. anti-NMDAR encephalitis), and subsequent neurological or psychiatric symptoms appearing after parasite clearance should not be automatically attributed to malaria recrudescence. Instead, after rigorously excluding parasitemia (via blood smear and PCR), AIE must be considered and systematically evaluated with antibody testing, neuroimaging, and CSF studies.

To improve early detection and treatment, enhancing access to testing for autoimmune antibodies (e.g., against NMDAR, VGKC-complex, AMPAR, and GABA_β_) is essential. Strengthening clinician awareness and laboratory capacity—particularly in rural areas—through reference collaborations or simplified assays can facilitate timely immunotherapy (e.g., corticosteroids or IVIG), potentially preventing long-term disability.

From a broader public health perspective, malaria-related immune dysregulation may extend beyond the nervous system. Case reports have described systemic autoimmune phenomena after malaria, including immune-mediated hemolysis/autoimmune hemolytic anemia (20–22), and antiphospholipid antibody–related abnormalities (23, 24). In contrast, evidence that malaria directly triggers new-onset systemic lupus erythematosus (SLE) remains limited; available literature more commonly suggests genetic or immunologic overlap and hypothesized exacerbation of pre-existing/subclinical autoimmunity rather than definitive post-malarial onset (25). Similarly, malaria has been associated with transient thyroid hormone alterations (26) and endocrine–immune interactions in experimental models (27), but convincing evidence for malaria as a trigger of autoimmune thyroiditis is currently lacking.

Integrating surveillance for post-malarial AIE into malaria control programs could help quantify incidence and identify risk factors. Post-treatment follow-up should also facilitate timely recognition of neuropsychiatric symptoms and other autoimmune sequelae after malaria, particularly in pediatric and young adult populations, in whom neurological sequelae after malaria are well documented (28).

## 4 Conclusion

This report presents the first antibody-confirmed case of anti-NMDAR encephalitis following malaria, highlighting that malaria can trigger AIE. Clinicians should consider this diagnosis in patients who develop new neuropsychiatric symptoms (e.g. psychosis, seizures, or cognitive decline) after parasite clearance, instead of attributing them solely to malaria relapse or residual PMNS. Therefore, rigorous exclusion of AIE requires appropriate antibody testing, neuroimaging, and CSF analysis. Early immunotherapy can significantly improve outcomes, underscoring the need to enhance access to diagnostics and increase clinical awareness in endemic regions to prevent diagnostic delays and long-term disability. Future studies should focus on identifying risk factors and establishing clear management guidelines for this severe post-infectious complication.

## 5 Limitations

This report is limited by its single-case design, which restricts generalizability. Although follow-up has been extended (≈8 months) with no clinical relapse and repeat serum anti-NMDAR IgG remaining negative, longer-term surveillance with serial CSF/serum antibody titers would better define relapse risk and immunological dynamics. In addition, the study is limited by its retrospective nature as serial serum samples prior to encephalitis onset were unavailable, and the autoantibody panel was restricted to clinically indicated targets. Larger studies with longer follow-up and broader immunoprofiling are warranted to confirm the association and clarify the underlying mechanisms.

## Data Availability

The raw data supporting the conclusions of this article will be made available by the authors, without undue reservation.

## Glossary

PMNS: Post-malarial neurological syndrome
DCA: Delayed cerebellar ataxia
AIDP: Acute inflammatory demyelinating polyneuropathy
ADEM: Acute disseminated encephalomyelitis
MS: Multiple sclerosis
NMOSD: Neuromyelitis optica spectrum disorder
CNS: Central nervous system
VGKC: Voltage-gated potassium channel
NMDAR: N-methyl-D-aspartate receptor
AIE: Autoimmune encephalitis
HSV: Herpes simplex virus
BBB: Blood-brain barrier
IFA: Indirect immunofluorescence assay
CBA: Cell-based assay
CSF: Cerebrospinal fluid
DP-T: Double-positive T cells
PfEMP1: Plasmodium falciparum erythrocyte membrane protein 1
IBPs: Immunoglobulin-binding proteins
ANA: Antinuclear antibody
EAE: Experimental autoimmune encephalomyelitis
Tregs: Regulatory T cells
TLR: Toll-like receptor
AMPAR: α-amino-3-hydroxy-5-methyl-4-isoxazolepropionic acid receptor
IVIG: Intravenous immunoglobulin;
